# Whole-brain structure–function coupling abnormalities in mild cognitive impairment: a study combining amplitude of low-frequency fluctuations and voxel-based morphometry

**DOI:** 10.3389/fnins.2023.1236221

**Published:** 2023-07-31

**Authors:** Rong Zhao, Pan Wang, Lin Liu, Fanyu Zhang, Peng Hu, Jiaping Wen, Hongyi Li, Bharat B. Biswal

**Affiliations:** ^1^MOE Key Laboratory for Neuroinformation, Center for Information in Medicine, School of Life Science and Technology, The Clinical Hospital of Chengdu Brain Science Institute, University of Electronic Science and Technology of China, Chengdu, China; ^2^The Fourth People’s Hospital of Chengdu, Chengdu, China; ^3^Department of Biomedical Engineering, New Jersey Institute of Technology, Newark, NJ, United States

**Keywords:** Alzheimer’s disease, amplitude of low frequency fluctuations, structure–function coupling, voxel-based morphometry, white matter

## Abstract

Alzheimer’s disease (AD), one of the leading diseases of the nervous system, is accompanied by symptoms such as loss of memory, thinking and language skills. Both mild cognitive impairment (MCI) and very mild cognitive impairment (VMCI) are the transitional pathological stages between normal aging and AD. While the changes in whole-brain structural and functional information have been extensively investigated in AD, The impaired structure–function coupling remains unknown. The current study employed the OASIS-3 dataset, which includes 53 MCI, 90 VMCI, and 100 Age-, gender-, and education-matched normal controls (NC). Several structural and functional parameters, such as the amplitude of low-frequency fluctuations (ALFF), voxel-based morphometry (VBM), and The ALFF/VBM ratio, were used To estimate The whole-brain neuroimaging changes In MCI, VMCI, and NC. As disease symptoms became more severe, these regions, distributed in the frontal-inf-orb, putamen, and paracentral lobule in the white matter (WM), exhibited progressively increasing ALFF (ALFF_NC_ < ALFF_VMCI_ < ALFF_MCI_), which was similar to the tendency for The cerebellum and putamen in the gray matter (GM). Additionally, as symptoms worsened in AD, the cuneus/frontal lobe in the WM and the parahippocampal gyrus/hippocampus in the GM showed progressively decreasing structure–function coupling. As the typical focal areas in AD, The parahippocampal gyrus and hippocampus showed significant positive correlations with the severity of cognitive impairment, suggesting the important applications of the ALFF/VBM ratio in brain disorders. On the other hand, these findings from WM functional signals provided a novel perspective for understanding the pathophysiological mechanisms involved In cognitive decline in AD.

## Introduction

Alzheimer’s disease (AD), a neurodegenerative disease primarily affecting the elderly, is marked by beta-amyloid plaques and neurofibrillary tau protein tangles (Rosales-Corral et al., 2012). There are no effective therapies that have been shown to change the pathophysiology or course of AD that cause the disease’s progression (Harrington, 2012). Depending on the symptoms displayed, AD can be classified as mild, moderate, or severe. Mild cognitive impairment (MCI), as an intermediate stage between normal aging and AD (Chandra et al., 2019), is mainly defined as neurocognitive impairment and does not meet the diagnostic criteria for dementia. Very mild cognitive impairment (VMCI) represents a transitional state between the cognitive changes of normal aging and the very early onset of dementia and is becoming increasingly recognized as a risk factor for Alzheimer’s disease (Chandra et al., 2019). Previous studies demonstrated that MCI is a significant risk factor for AD and that approximately 10 to 15% of MCI will eventually progress to AD (Petersen et al., 2001; Ronald et al., 2001). This is the reason why early assessment of MCI may help researchers discover the pathophysiological mechanisms of AD and provide new information for better treatment in the future (Weller and Budson, 2018; Gupta et al., 2020).

Neuroimaging research has been crucial in the clinical diagnosis of MCI and AD, and magnetic resonance imaging (MRI) modalities have proven useful in the identification of biomarkers for AD and MCI pathology (McKhann et al., 2011). AD progression has been tracked with structural imaging techniques to demonstrate that atrophy spreads to the medial temporal lobe, parahippocampal, fusiform gyri, and orbitofrontal cortex (Li et al., 2011). Atrophy in AD subjects is distributed in the frontal, parietal, and temporal lobes, whereas atrophy in MCI subjects is in the frontal and temporal lobes (Guo et al., 2010). The aforementioned research on AD has focused on gray matter (GM) or white matter (WM) structures.

Blood oxygenation-level-dependent (BOLD) signals have primarily focused on the GM but are rarely reported on the WM (Mazerolle et al., 2020; Abramian et al., 2021). Gawryluk and colleagues comprehensively demonstrated the possibility that non-invasive fMRI techniques could detect the WM functional signals in the human brain, which laid a good foundation for the study of WM functional images in the future (Gawryluk et al., 2014). The WM BOLD signal has recently been shown to play an important role in understanding cerebral intrinsic activity (Ji et al., 2017; Ding et al., 2018; Li et al., 2019; Wang et al., 2020). Ji et al. (2017) demonstrated that the BOLD signal in the white matter varies with physiological states, closely correlating with structural features. Ding et al. (2018) have found that BOLD signals in specific WM tracts are functionally correlated with specific GM regions during different tasks. The BOLD signals reflect changes in cerebral blood flow and volume and are used to study dynamic representations of brain activity (Chen and Pike, 2009; Wang et al., 2021). Blood flow and blood volume in WM are approximately 25% of those in GM (Rostrup et al., 2000; Helenius et al., 2003). In both GM and WM, resting-state BOLD fluctuations have similar low-frequency (0.01–0.1 Hz) signal power (Gore et al., 2019; Wang et al., 2022). Changes in BOLD signals in the WM reflect a distinct response to neuronal activity because they vary with baseline neural activity (Abramian et al., 2020). Recent studies have shown that sensory stimulation evokes reliable BOLD responses along with a subset of fiber pathways (Huang et al., 2018). After brief stimulation, WM bundles also show a transient signal response similar to GM but not identical to the characteristics of the hemodynamic response function of GM (Wu et al., 2017). Thus, there is increasing evidence that WM exhibits resting-state fluctuations and a transient signal response (Ding et al., 2016). These studies provide evidence that BOLD signals in WM are associated with functional activity in the human brain and deserve more attention from the neuroimaging community (Gore et al., 2019).

The amplitude of low-frequency fluctuation (ALFF) has been used to measure the amplitude of low-frequency oscillations in resting-state fMRI, and it is defined as the total power within the frequency range from 0.01 to 0.1 Hz (Han et al., 2011; Di et al., 2013; Yang et al., 2018). The BOLD signals of resting-state fMRI are thought to represent spontaneous brain activity and are a good indicator of the regional intensity of spontaneous variations of ALFF (Fox and Raichle, 2007). Previous research (Han et al., 2011; Chen et al., 2015; Wang et al., 2016) has suggested that the ALFF measure can be used as a predictor for disease states such as MCI, tinnitus, and depression. A propensity for ALF*F* values to decrease as AD progresses can be seen in the right median and paracingulate gyri, bilateral inferior cerebellar lobes (which extend to the posterior cerebellum), bilateral precuneus, and the right anterior cingulate (Yang et al., 2018; Luo et al., 2020).

One potential area for indicators is structure-based neuroimaging research, with voxel-based morphometry (VBM) in particular serving as a well-established technique for identifying brain atrophy. In early AD prodromal stages, compared to healthy senior controls, VBM studies have found a high similarity in the olfactory cortex and hippocampus (Pennanen et al., 2004; Vasavada et al., 2015). Several atrophies in the temporal, parietal, posterior cingulate, frontal cortices, insula, and Rolandic eyes have been documented during the senile stage of AD (Duarte et al., 2006). Lower GM volume was demonstrated by Roquet et al. (2017) in the anterior cuneus, frontal lobe, insula, and extratemporal lobe. Axonal atrophy and demyelination were the findings of two early studies that described alterations in WM macro- and microstructure in AD (Brun and Englund, 1986). Additionally, earlier research showed that ALFF and its association with fractional asymmetry in WM supported the idea that structure–function coupling is a fundamental organizing principle in healthy subjects (Honey et al., 2009; Wang et al., 2015).

In the past, functional activity in predetermined brain areas has been examined using a mix of structural and functional brain imaging. The integration of functional and structural MRI, for instance, may provide complementary information to improve the diagnosis of MCI relative to either alone (error rate: 6% with both versus 15% with fMRI alone and 35% with sMRI alone), according to a classification study (Kim and Lee, 2013). In order to comprehend the aberrant changes in brain diseases, it was essential to combine the anatomical and functional information from neuroimaging techniques.

The current study aimed to examine the functional and structural changes within the whole brain in AD by combining ALFF and VBM analysis. First, we separately investigated the abnormal ALFF and VBM within the whole brain. Then, the ALFF/VBM ratio would be adopted here as the regional-level measure. In addition, we analyzed the studies of structural-functional association *via* direct ALFF–VBM comparisons. Furthermore, we analyzed the abnormal changes in whole-brain ALFF–VBM coupling in the MCI and VMCI groups. Finally, we estimated the relationships between the above abnormal regions and the clinical manifestations of AD.

## Materials and methods

In this study, we used 243 subjects in the publicly available OASIS-3 dataset^1^, with 53 MCI, 90 VMCI, and 100 normal control (NC) subjects. We collected resting-state functional images and T1-weighted anatomical images for each subject. All data were collected from the University of Washington Knight AD Research Center in various studies conducted over the past 15 years. Written informed consent was obtained from all participants before collecting fMRI data. The dementia spectrum status of all patients was assessed using the Clinical Dementia Rating (CDR) scale (Morris, 1993). According to the CDR, all AD patients were divided into NC (CDR = 0), VMCI (CDR = 0.5), and MCI (CDR = 1), respectively. The severity of cognitive impairment was assessed based on the Mini-Mental State Examination (MMSE; Folstein et al., 1975). Detailed demographic and clinical information is described in Table 1.

**Table 1:** Demographic and clinical characteristics of the subjects.

Characteristics	NC (*N* = 100)	VMCI (*N* = 90)	MCI (*N* = 53)	*p* Value
Age	74.41 ± 8.227	74.76 ± 7.668	75.89 ± 8.554	*p* = 0.4940^a^
Gender (M/F)	50/50	48/42	33/20	*p* = 0.3471^b^
Education	14.32 ± 1.757	14.59 ± 3.016	14.75 ± 3.204	*p* = 0.8438^a^
Mean FD	0.2825 ± 0.1504	0.3022 ± 0.1756	0.3148 ± 0.1489	*p* = 0.2808^a^
MMSE	28.86 ± 1.318	25.99 ± 2.882	22.08 ± 4.057	*p* < 0.001^a^
Handedness (L/R)	0/100	0/90	0/53	

M = male patient; F = female patient; L = left; MMSE = mini-mental state examination; FD = framewise displacement; MCI = mild cognitive impairment; NC = normal controls; R = right; VMCI = very mild cognitive impairment.

a One-way ANOVA (using a nonparametric test).

b Chi-squared.

### Data acquisition

All of the OASIS MRI datasets used in this study were imported on a 3-T Siemens Trio Tim scanner. During the scanning session, each subject was asked to keep their eyes closed with no significant head movement and not to fall asleep or think systematically. The resting-state functional imaging acquisition parameters were as follows: voxel size = 4 × 4 × 4 mm^3^, echo time = 27 ms, repetition time = 2.2 s, flip angle = 90°, slice thickness = 4 mm, number of time points = 164. The high spatial resolution three-dimension T1-weighted anatomical images were acquired with the following parameters: voxel size = 1 × 1 × 1 mm^3^, echo time = 3.16 ms, repetition time = 2.4 s, flip angle = 8°, slice thickness = 1 mm.

### Data preprocessing

#### Functional images

The functional MRI dataset was preprocessed using the Data Processing Assistant for Resting-State fMRI^2^ and SPM12 software^3^ toolkits. The preprocessing steps included: discarding the first 5 volumes, correcting for head motion-related signal changes, and co-registering individual T1-weighted structural images to the mean functional image. Each structural image was segmented into GM, WM, and cerebrospinal fluid (CSF) using the DARTEL segmentation algorithm. Linear trends were removed to correct for signal drift. The mean signal from CSF and 24 rigid body motion parameters (six head motion parameters, six values at previous time points of six head motion parameters, and the 12 corresponding squared items) were regressed from the functional images. Scrubbing using motion “spikes” was performed as separate regressors, identified by framewise displacement (FD) greater than 1 mm to further reduce the effect of head motion. The head motion scrubbing regressors were used in this study as they have been shown to be effective in eliminating the effect of head motion at the spike on the signal without changing the correlation values (Power et al., 2012). Temporal filtering was performed in the low-frequency range of 0.01–0.1 Hz. To avoid mixing of WM and GM signals, the functional images were minimally spatially smoothed (4 mm full-width half-maximum [FWHM], isotropic) separately in the WM and GM templates for each subject. The WM and GM voxels were identified by using the segmentation results from each subject (using a threshold of 0.5 in the SPM12’s tissue segmentation). Following smoothing, the functional image was normalized to the Montreal Neurological Institute (MNI) space of 3 × 3 × 3 mm^3^.

### Creation of group-level WM and GM masks

To create WM and GM masks at the group level, we used the T1 segmentation images to identify the binarized WM and GM masks based on the maximum probability of each voxel within the whole brain for each subject. Subsequently, we averaged these WM and GM masks across all subjects. The group-level WM mask was defined by using the following two steps: (1) a threshold of more than 60% of subjects was used to determine the binarized anatomical mask, in line with previous studies on WM functional signals (Mazerolle et al., 2020; Wang et al., 2022); (2) the voxels of the anatomical mask were then selected and recognized as the group-level WM mask in more than 80% of subjects in the functional data. Finally, to exclude the influence of deep brain structures, we removed subcortical areas using the Harvard-Oxford atlas. In addition, for the group-level GM mask, a loose threshold > 20% was used to identify the GM mask that contains almost all GM voxels. The voxels in the resulting mask were then selected, and the group-level GM mask was defined using voxels that were present in more than 80% of subjects in the functional data.

### Structure–function coupling analysis

#### Amplitude of low-frequency fluctuations analysis

For the calculation of voxel-wise power associated with low-frequency fluctuations, whole-brain ALFF was calculated for each subject using the DPABI package (DPABI_V6.0_210501).^4^ After preprocessing the functional images, the time series of each voxel was filtered (band-pass, 0.01–0.1 Hz) to eliminate the effects of high-frequency drift and low-frequency noise (Biswal et al., 1995; Power et al., 2012). For each voxel, the time series was converted to the frequency domain by a fast Fourier transform. The ALFF in a given voxel is defined as the average square root value of the power spectrum in the frequency interval 0.01–0.1 Hz (Zang et al., 2012).

#### Voxel-based morphometry analysis

Anatomical images were analyzed using an optimized computational anatomy toolbox with the CAT 12 package in MATLAB.^5^ The procedure is relatively straightforward and involves spatially normalizing high-resolution images from all the subjects in the study into the same stereotactic space. GM and WM are then segmented from the spatially normalized images, and the images are smoothed. Voxel-wise parametric statistical tests comparing the smoothed GM and WM images from the three groups are performed. Corrections for multiple comparisons are performed using Gaussian random field (GRF) theory.

#### Amplitude of low-frequency fluctuations and voxel-based morphometry coupling analysis

After calculating the ALFF and VBM as described above, we can obtain the ALFF map for each subject and the corresponding VBM map. For each subject, we co-registered the ALFF map with the corresponding VBM map, and then the voxel value of ALFF was divided by that of VBM for each voxel.

### Statistical analysis

A one-way ANOVA analysis was performed using the DPABI software. We performed it on the ALFF maps within group-level GM masks among the NC, VMCI, and MCI groups while controlling for the effects of age, gender, and education. We used GRF correction (voxel-level: *p* < 0.001, cluster level: *p* < 0.01) to estimate the statistical significance. Subsequently, the above abnormal clusters were obtained for *post hoc* analysis and were compared using the two-sample t-test with age, gender, and education as covariates (two-tailed, *p* < 0.05, Bonferroni-corrected for multiple comparisons (*p* < 0.05/*n*), *n* is the number of abnormal regions). For abnormal ALFF in the WM, we repeated the above analysis process. In addition, using the same statistical analysis process, we investigated the abnormal VBM and function–structure coupling in the GM/WM, respectively.

## Results

### Abnormal ALFF in the GM/WM

**Figure 1:**
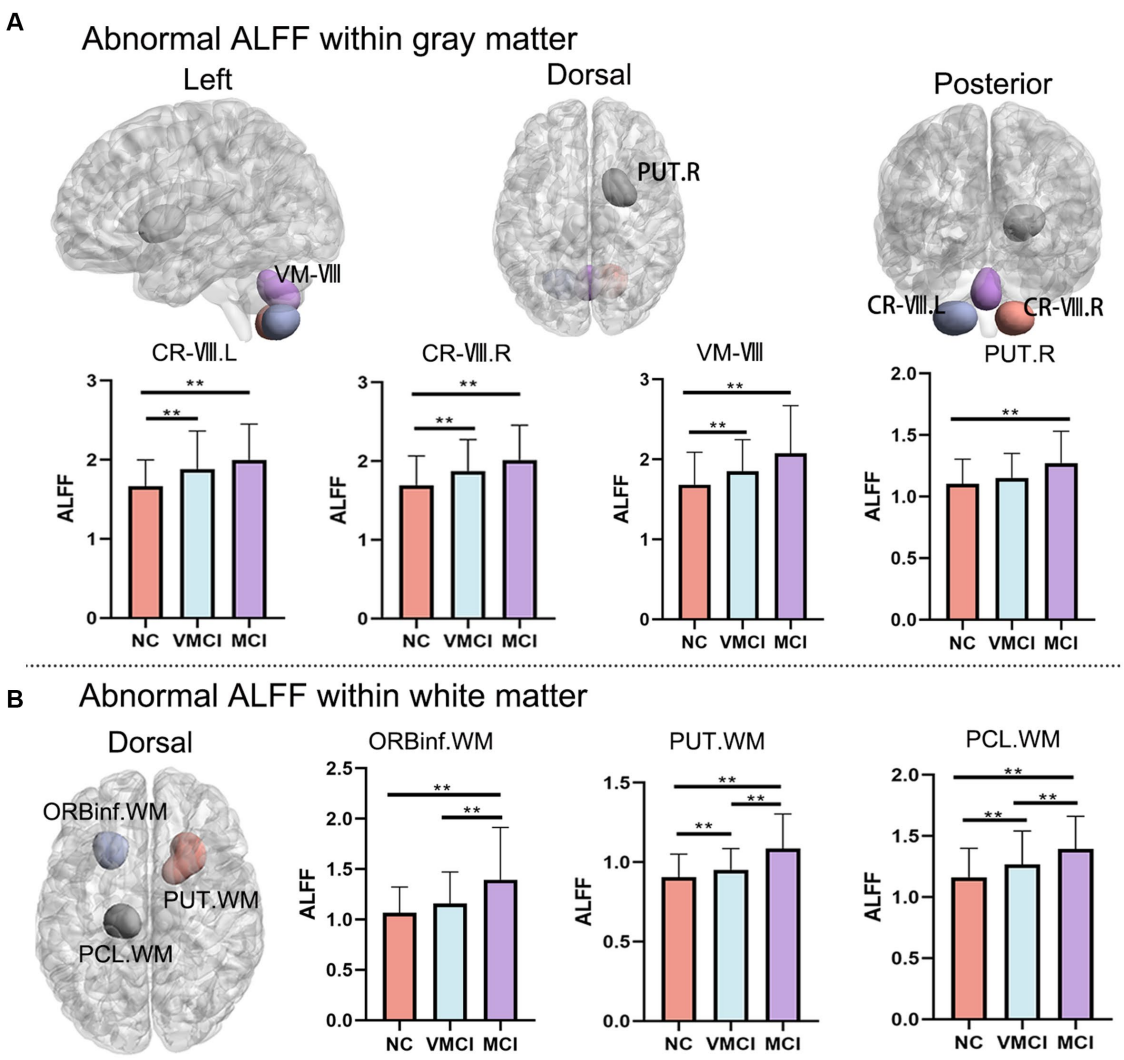
Whole-brain ALFF changes. Brain map shows the abnormal clusters within GM and WM, respectively. Histogram shows the post-hoc analysis results between NC, VMCI and MCI. **(A)** and **(B)** represent the ALFF changes within GM and WM, respectively. The statistic significant level is performed with GRF correction (voxel-level: *p* < 0.001, cluster-level: *p* < 0.01).

Using the ALFF measure, we identified seven abnormal areas distributed in the GM and WM (Figure 1; Table 2). Specifically, compared to NC, patients with MCI and VMCI showed significantly increased ALFF in the GM distributed in Cerebellum-VIII-L (CR-VIII.L), Vermis-VIII (VM-VIII). and Cerebellum-VII-R (CR-VIII.R), but no difference between VMCI and MCI. Moreover, compared to NC, we found abnormally increased ALFF distributed in the putamen (PUT.R) in MCI.

**Table 2:** The abnormal ALFF and structure–function coupling regions in the GM and WM.

Regions	MNI coordinates	Peak value (*F* value)	Cluster size	VMCI vs. MCI (*U*/*P* value)	VMCI vs. HC (*U*/*P* value)	MCI vs. HC (*U*/*P* value)
Abnormal ALFF in the GM						
Cerebellum-VII-L	−21,-66,-54	12.09	30	1965/0.0795	3295/0.0014^*^	1,483/<0.0001^*^
Cerebellum-VII-R	15,-60,-57	12.18	28	1932/0.0584	3311/0.0016^*^	1,549/<0.0001^*^
Vermis-VII	0,-69,-42	11.38	24	1861/0.0283	3247/0.0009^*^	1,433/<0.0001^*^
Putamen-R	21,9,3	13.67	13	1801/0.0144	3741/0.0449	1,600/<0.0001^*^
Abnormal ALFF in the WM						
Frontal-Inf-Orb-L	−15,-21,45	15.31	47	1589/0.0008^*^	3599/0.0171	1,382/<0.0001^*^
Putamen-R	24,21,0	14.30	39	1494/0.0002^*^	3542/0.0112^*^	1,296/<0.0001^*^
Paracentral-Lobule-L	−24,21,-12	15.69	21	1696/0.0038^*^	3378/0.0029^*^	1,302/<0.0001^*^
Abnormal structure–function coupling ratio in the GM						
Cerebellum-VII-R	21,-60,-57	9.12	16	2222/0.4983	3,005/<0.0001^*^	1887/0.0033^*^
ParaHippocampal-R	21,-9,-6	21.65	146	1747/0.0074^*^	2064/<0.0001^*^	600/<0.0001^*^
Hippocampus-L	−24,-12,-6	20.61	112	1625/0.0014^*^	2,211/<0.0001^*^	671/<0.0001^*^
Abnormal structure–function coupling ratio in the WM						
Cuneus-R	15,-84,18	10.99	17	1984/0.0942	3209/0.0006^*^	1,480/<0.0001^*^
Frontal Lobe	−2,-21,45	13.82	15	1801/0.0144^*^	3526/0.0099^*^	1,390/<0.0001^*^

^*^Represents a significant level with statistical correction (Bonferroni-corrected for multiple comparisons (*p* < 0.05/*n*), *n* is the number of abnormal regions).

In addition, we observed abnormally increased ALFF in the WM, which was similar to the increased pattern in GM (Figure 1B). In detail, compared to NC, patients with MCI and VMCI showed significantly increased ALFF in Putamen-R (PUT-WM) and Paracentral-Lobule-L (PCL-WM). We also found significant differences in the above WM regions between the MCI and VMCI groups. In addition, we observed increased ALFF in the frontal-inf-orb-L (ORBinf-WM) area in the MCI group compared to the comparison, but no difference between VMCI and NC.

### Abnormal VBM in the GM/WM

**Figure 2:**
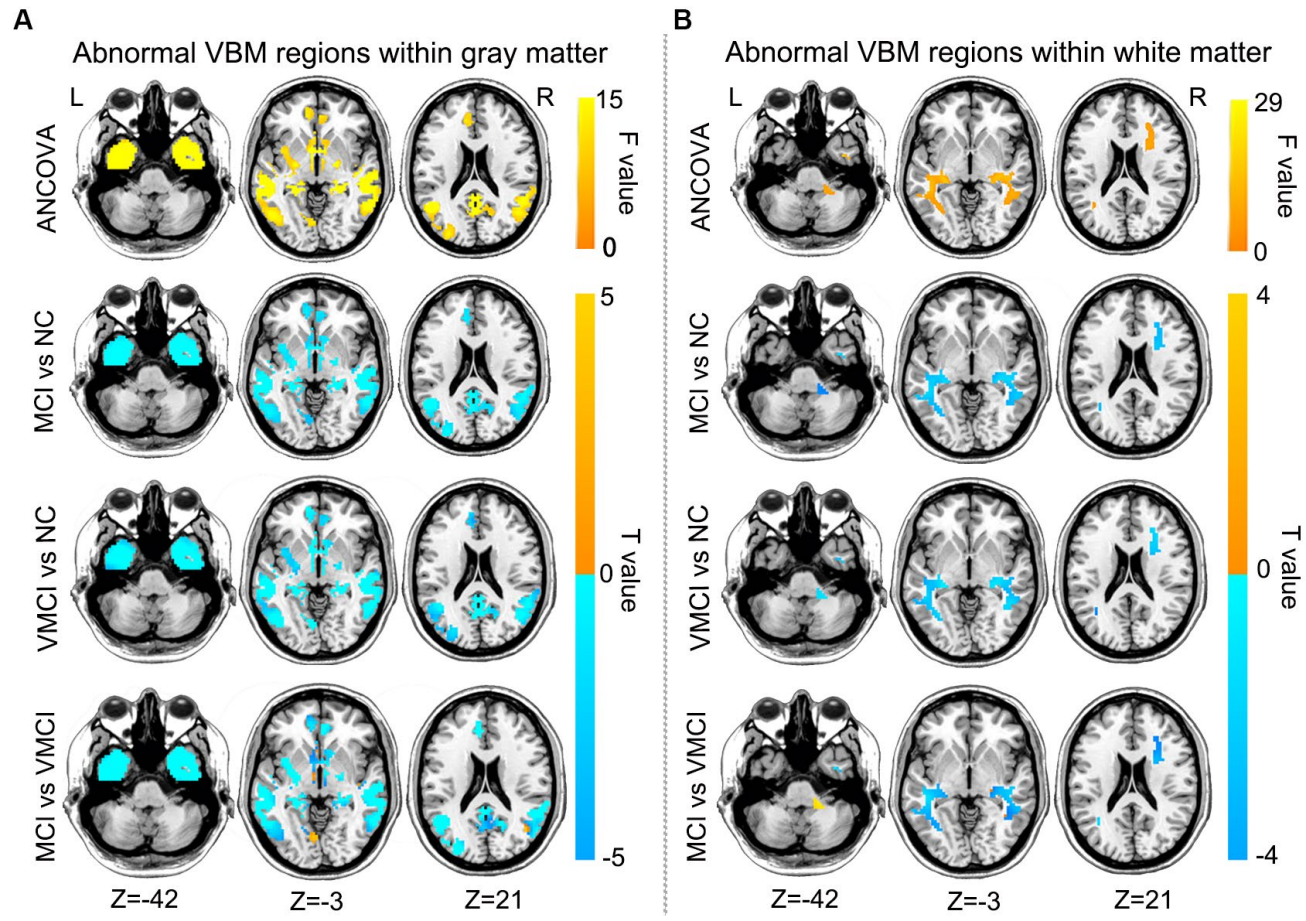
Brain regions with abnormal VBM between patient groups and NC subjects, GM **(A)** and WM **(B)** based on *post-hoc T*-tests. Blue and yellow colors denote decreased and increased VBM. The yellow arrow shows the corresponding brain region. The color bars indicate the *T*-value (GRF correction, voxel-level: *p* < 0.001, cluster level: *p* < 0.01).

To estimate the structural abnormality in the early stages of AD, we performed the VBM analysis in the present study. Compared to NC, patients with MCI and VMCI showed significantly reduced GM volume distributed in the frontal lobe, temporal lobe, cerebellum, hippocampus, fusiform gyrus, and precuneus. In addition, compared to the VMCI group, MCI showed significantly reduced volume in the bilateral precentral gyrus and postcentral gyrus. Furthermore, in comparison to VMCI, MCI showed mild GM atrophy distributed in the middle temporal lobe and medial frontal lobe (Figure 2A). We also found that the MCI and VMCI groups showed significantly decreased volume in the WM, mainly distributed in the right insula, rolandic operculum, and superior temporal lobe (Figure 2B).

### Abnormal ALFF/VBM coupling results in the GM/WM

**Figure 3:**
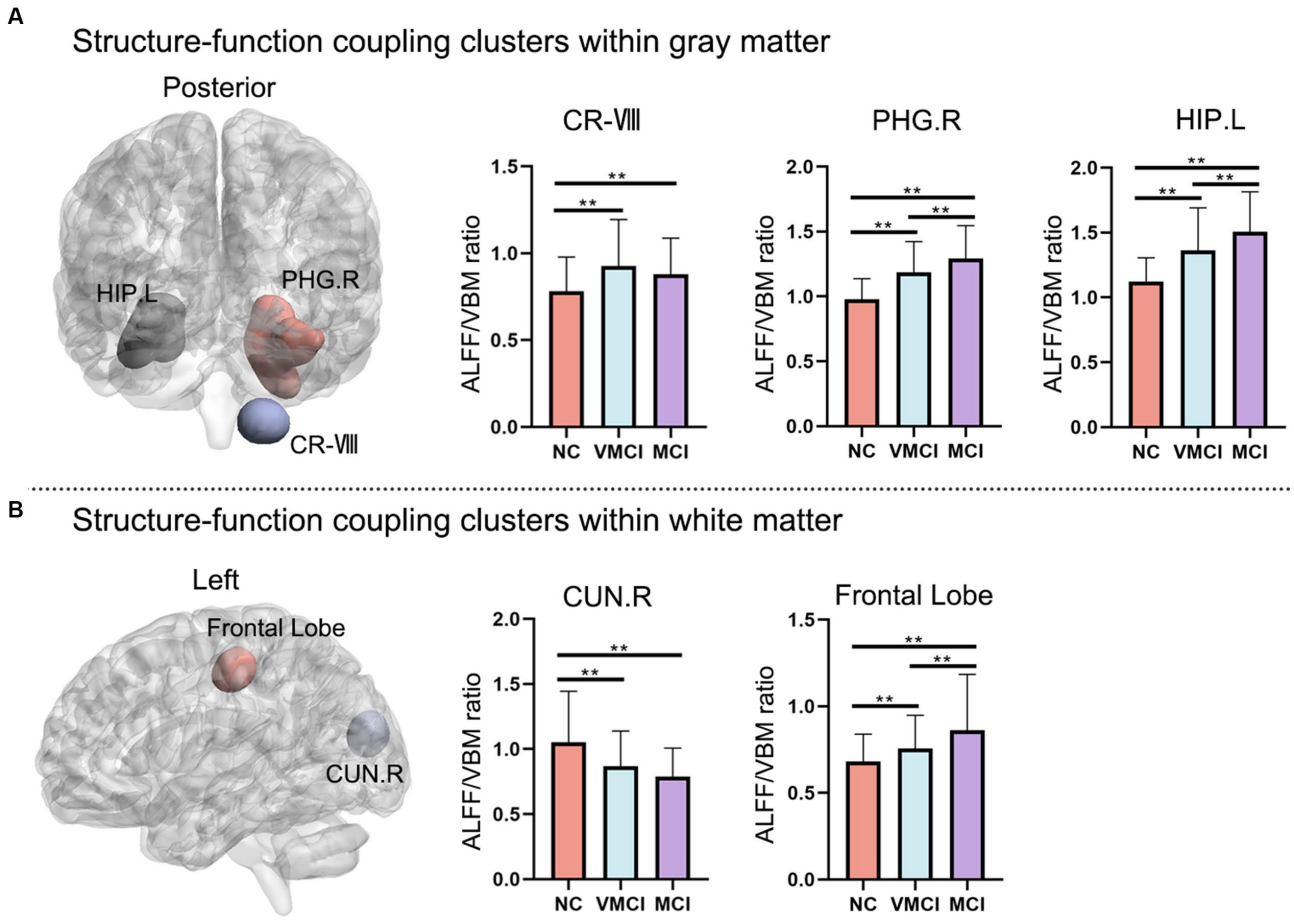
Abnormal regions of structure–function coupling (ALFF/VBM ratio) indicators. **(A)** and **(B)** represent the abnormal regions of structure–function coupling within the GM and WM, respectively. The left side shows the abnormal brain regions and the right side shows the histogram with *post-hoc* analysis results. (GRF correction, voxel-level: *p* < 0.001, cluster level: *p* < 0.01).

Using the structure–function coupling measure, we found five abnormal clusters in three groups (Figure 3; Table 2). In detail, compared to NC, patients with VMCI showed abnormally increased function–structure coupling in Cerebellum-VIII-R (CR-VIII), but there was no difference between VMCI and MCI. In addition, we found an abnormally increased ALFF/VBM ratio in ParaHippocampal-R (PHG.R) and hippocampus (HP.L) in patients with MCI and VMCI compared to NC, also in the comparison of MCI and VMCI (Figure 1A). The WM results also showed that the averaged ALFF/VBM ratio values in the fusiform gyrus (FG-WM) and cuneus (CUN-WM) were abnormally higher in the patient groups than in the NC (Figure 1B).

### Associations between altered brain regions and clinical characteristics

**Figure 4:**
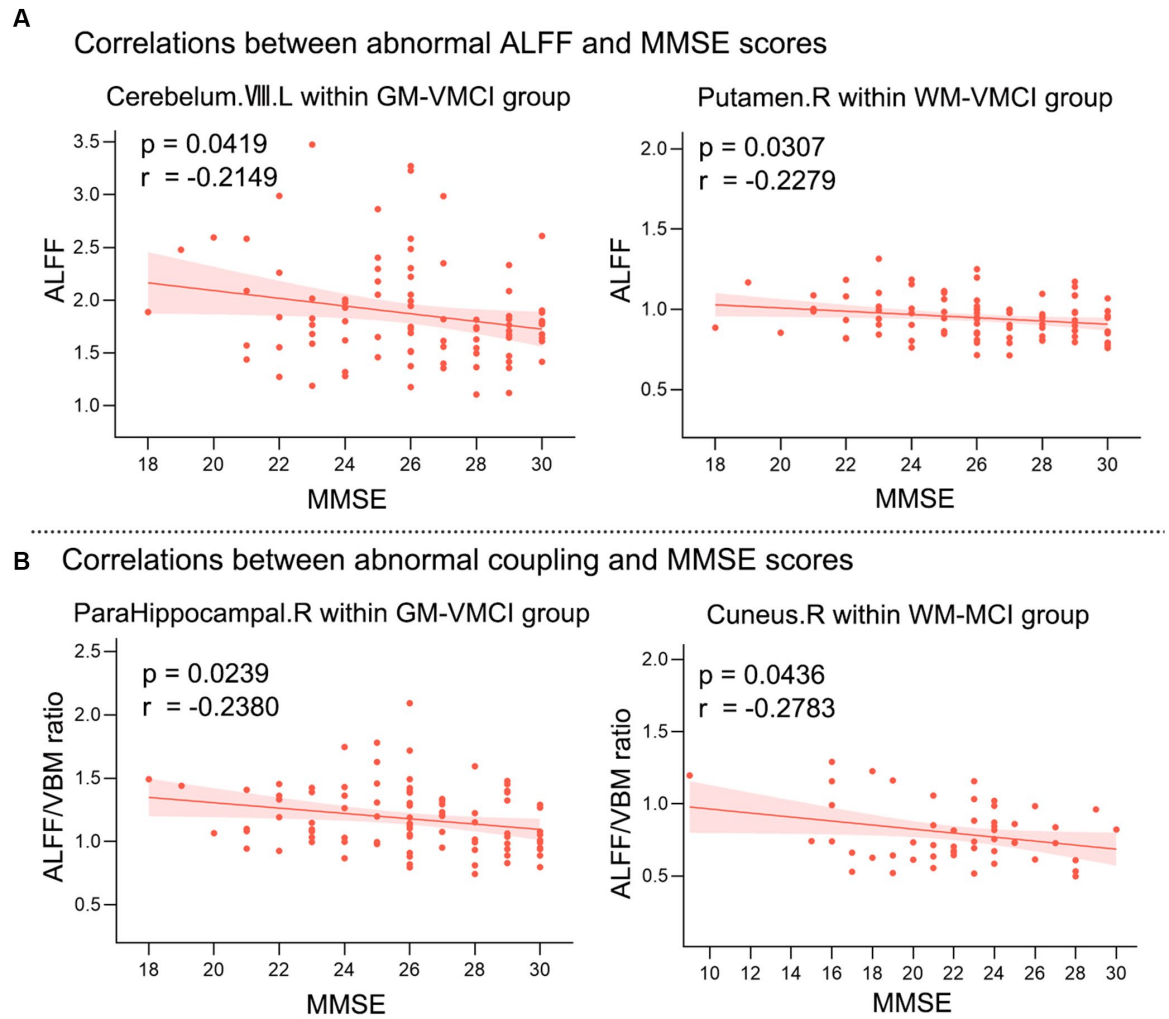
Relationships between averaged abnormal clusters and MMSE scores. **(A)** shows the correlations between abnormal ALFF values and MMSE scores. **(B)** shows the correlations between abnormal coupling values and MMSE scores. X-axis = MMSE; Y-axis = ALFF or ALFF/VBM ratio.

Pearson’s correlation analysis was performed to estimate the relationships between abnormal clusters and MMSE scores in MCI and VMCI (Figure 4). We found negative correlations between the averaged ALFF values distributed in the CR-VIII and PUT-WM and MMSE scores in the VMCI group (CR-VIII: *p* = 0.0419, *r* = −0.2149; PUT-WM: *p* = 0.0307, *r* = −0.2279). In addition, we also observed negative correlations between the averaged coupling values in PHG.R and MMSE scores in the VMCI group (*p* = 0.0239, *r* = −0.238), and between the averaged coupling values in CUN-WM and MMSE scores in the MCI group (*p* = 0.0436, *p* = −0.2783).

## Discussion

In this study, we investigated the abnormal neuroimaging changes in the early stages of AD by combining whole-brain ALFF and VBM. We adopted novel measures (ALFF/VBM ratio) to investigate the whole-brain structure–function coupling abnormalities. For the MCI and VMCI groups, significant differences in ALFF were observed in VM-VIII, PUT.R, CR-VIII in the GM, and ORBinf-WM, PUT-WM, and PCL-WM. Additionally, we found the structure–function coupling clusters distributed in CR-VIII, PHG.R, and HP.L in the GM, and CUN.R and the frontal lobe in the WM. More importantly, as the typical focal areas in AD, PHG.R exhibited a negative correlation with the severity of cognitive impairment, indicating the important applications of the ALFF/VBM ratio in brain disorders. Findings from WM functional signals provided a novel perspective for understanding AD pathophysiology.

Using ALFF values from resting-state fMRI signals, we estimated the VMCI- and MCI-related changes in intrinsic and spontaneous brain activity. We found that there were widespread differences in ALFF values among the VMCI, MCI, and healthy elderly patients in the different brain regions of the frontal, temporal, and parietal lobes and in subcortical areas. A previous study showed decreased ALFF in the left hippocampus and PCC (Sorg et al., 2007; Wang et al., 2011). Different ALFF values in the GM were observed in the PUT.R, and VM-VII among the NC, VMCI, and MCI groups. Also, there was a significant abnormality in the PUT.R. The abnormal ALFF values in the PUT.R were significantly attributed to the memory decline over time and more severe AD pathology. Previous studies have shown that the PUT, as part of the striatum, is active during working memory tasks (Dahlin et al., 2008) and probabilistic learning tasks (Bellebaum et al., 2008). Cross-sectional studies have reported disrupted functional connectivity involving the PUT in individuals with MCI compared to their healthy counterparts (Teipel et al., 2007; Bai et al., 2011; Han et al., 2011). Our findings of ALFF changes in PUT, in line with two previous studies, likely strengthen the evidence for memory decline over time and more severe AD pathology. In the first study, it was reported that atrophy of the PUT but not the caudate was significantly associated with cognitive decline in patients with probable AD (de Jong et al., 2008). In the second study, the other reported abnormally high perfusion in the PUT but not the caudate in MCI compared to AD patients (Alexopoulos et al., 2012). Thus, our finding of abnormal ALFF in the PUT, together with our recognition, suggests that the PUT is an important brain region that deserves our attention. In addition, our study also found that ALFF in cerebellar VM-VII is abnormal in three groups. Such an approach was published in the 1990s, when research demonstrated that patients with cerebellar damage had cognitive deficits (Schmahmann, 1991). More recently, much evidence has shown that the cerebellum affects not only visuospatial and verbal functions and declarative memory but also more complex behavioral regulatory processes, namely executive function (Mak et al., 2016; Myers et al., 2017; Beuriat et al., 2020; Liu et al., 2021). A previous study demonstrated that patients with cognitive impairment had reduced fractional ALFF values in the cerebellar regions closely related to cognitive function compared to HC (Fan et al., 2019). The present study found that the widely distributed cerebellar ALFF changed with the severity of cognitive impairment, suggesting the important role of the cerebellum in normal cognitive functional activity. We also found abnormalities in CR-VIII. L, CR-VII. R in the GM between NC and the other two disease groups. According to previous studies, the cerebellum is involved in motor, balance, and cognitive functions (Wagner and Luo, 2020), so we think that cerebellar abnormalities are linked to the dysfunction in MCI and VMCI.

As for the WM results, three clusters (ORBinf-WM, PUT-WM, and PCL-WM) were consistently significantly abnormal across the three groups. We analyzed significant differences in ALFF values. ORBinf-WM is a part of the orbitofrontal cortex (OFC) and prefrontal cortex regions in the frontal lobe. It shares extensive connections with other cortices such as motor, primary sensory, and association cortices, the hippocampus, the amygdala, and other areas such as olfactory, auditory, and periaqueductal grey areas. The extensive connections of ORBinf-WM indicate that ORBinf-WM has extensive and complex functions (Cavada et al., 2000; Kringelbach, 2005; Barbas, 2007). Therefore, the abnormal differences in ALFF in ORBinf-WM of our MCI and VMCI patients imply that they might lead to a series of clinical manifestations such as poorer cognitive, behavioral, mental, and mood performance. Especially in the MCI, it will manifest in hallucinations, psychosis, depression, anxiety, apathy, sleep problems, and sexual desire dysfunction. The PCL-WM in the WM regions is distributed in the anterior portion of the paracentral lobule involved in the frontal lobe, which is often referred to as the supplementary motor area. The posterior portion is considered part of the parietal lobe and deals with the somatosensory system of the distal limbs^6^, demonstrating a potential relationship between associative memory and cognitive disorders. It can be inferred that as the disease develops, neural damage strengthens these three brain regions in the WM. VBM is a reliable method to compare typical brain regions in GM and WM between different groups of patients. Results among the three groups showed more GM atrophy in parts of the parietal and temporal lobes, PUT, insula, parahippocampal gyrus, and frontal lobe of both hemispheres. The above results are consistent with the research of Michaela Defrancesco et al. who reported disrupted WM integrity in the frontal, temporal, and parietal lobes and in subcortical structures in patients with MCI. In addition, AD subjects showed reduced GM density in the frontal lobe, left parietal lobe, left putamen, left insula, left temporal lobe, and right parahippocampal gyrus in comparison to MCI patients (Defrancesco et al., 2014). MCI compared to VMCI, showed mild GM atrophy in areas such as the middle temporal lobe and medial frontal lobe, both of which are mainly involved in verbal memory and naming. In addition, atrophy of these brain regions and limbic system structures has been reported in MCI patients years before conversion to AD in numerous previous volumetric MRI studies (Ferreira et al., 2011). Regions of the default mode network (DMN), hippocampus, insula, and middle temporal gyrus showed significant GM loss in MCI, suggesting structural impairment in patients with MCI (Tisserand et al., 2004). In WM, our results are consistent with several previous findings showing disruption of WM integrity in frontal, temporal, parietal, and subcortical structures in patients with MCI and VMCI (Wang et al., 2006; Liu et al., 2013; Bao et al., 2022). These outcomes suggest that prefrontal and temporal cortical regions are of particular relevance in cognitive decline in healthy elderly individuals (Defrancesco et al., 2014).

As a major AD symptom seen in clinics (Jaroudi et al., 2017), the PHG and PH regions exhibited abnormally increased structure–function coupling values in the VMCI and MCI groups (Figure 3). The role of the parahippocampal gyrus in AD has long been known, and our research discovered an abnormal ALFF/VBM ratio in the PHG.R and HP.L between MCI and VMCI patients, indicating that the coupling technique may have some validity (Van Hoesen et al., 2000). The medial temporal lobe, which is primarily made up of the hippocampus, perirhinal and entorhinal cortices, and parahippocampal cortex involved in episodic memory, is where the early neuropathological changes that are characteristic of AD (de Leon et al., 1997; Dickerson and Eichenbaum, 2010; Eichenbaum et al., 2012). The PHG, which includes the middle temporal gyrus (MTG), angular gyrus, posterior cingulate cortex/precuneus/cuneus (Pcc/Pcu/Cun), and medial frontal cortex, is the area of the brain most commonly impacted by AD (MPFC; Greicius et al., 2004; Buckner et al., 2008). Additionally, there was a strong correlation between the structure–function coupling values of the PHG.R cluster and HP.L and our clinical heterogeneity in the MMSE results. It has been proposed that the DMN participates in activities that are inwardly directed, such as retrieving autobiographical memories and planning for the future (Chen et al., 2015). The PHG mainly receives direct associative input from the posterior associative brain areas, which are a component of the DMN. It then strongly projects to the hippocampus, a hub region in the episodic memory network that is located in the caudal two-thirds of the entorhinal cortex (Lavenex and Amaral, 2000; Witter et al., 2000; Spaniol et al., 2009; Chen et al., 2014). Understanding the functions of the PHG and the brain regions involved in the DMN in impaired episodic memory in AD/MCI is strengthened by knowledge of disease severity-related changes in connectivity between them. CUN-WM and FG-WM were both found to have distinct abnormal brain regions compared to NC and the other two cases. This was also seen in the ALFF and VBM findings of our study, adding to the mounting evidence that this area of the brain is abnormal in the early stages of AD.

## Limitations

Our study has four limitations that need to be considered. First, we did not include the subjects’ vascular risk factors (such as hypertension, hyperlipidemia, diabetes, obesity, excessive smoking, and mental stress) in the covariates of the regression analysis. The vascular risk factors may influence cognitive function in the elderly, which may be related to AD (Chong et al., 2017). Second, we have only analyzed rs-fMRI data. In future studies, the combination of multimodal neuroimaging and biological information may provide a more comprehensive understanding of the progression patterns in AD. Third, our study lacked extensive neuropsychological testing, and more exhaustive neuropsychological testing will be necessary to examine more cognitive aspects of patients to further explore the underlying mechanisms in the brain. Finally, we found that the clinical correlation coefficients between abnormal clusters and the MMSE in Figure 4 did not pass the correction for multiple comparisons. Further studies are needed to replicate the correlation plot results.

## Conclusion

This study investigated the changes in whole-brain neuroimaging in the early stages of AD by combining ALFF and VBM measures and further adopted the novel coupling index (ALFF/VBM ratio) to investigate the whole-brain structure–function coupling abnormalities. Compared to NC, MCI, and VMCI groups, significant ALFF differences were distributed in the VM-VIII, PUT.R, and CR-VIII in the GM, ORBinf-WM, PUT-WM, and PCL-WM. Moreover, we observed the coupling differences distributed in the CR-VIII, PHG.R, and HP.L in the GM, and CUN.R and frontal lobe in the WM, which were closely associated with MMSE. The above findings demonstrated the important applications of the ALFF/VBM ratio in brain disorders and provided a novel opinion for understanding the pathophysiology of AD from the perspective of WM functional signals.

## Data availability statement

The original contributions presented in the study are included in the article/Supplementary material, further inquiries can be directed to the corresponding authors.

## Ethics statement

The studies involving human participants were reviewed and approved by University of Washington Knight AD Research Center and School of Life Science and Technology, University of Electronic Science and Technology of China. The patients/participants provided their written informed consent to participate in this study. Written informed consent was obtained from the individual(s) for the publication of any potentially identifiable images or data included in this article.

## Author contributions

RZ performed all data analysis and wrote the whole manuscript. RZ, PW, and BB contributed to the study design and sorting of the experimental data, modifying the manuscript. LL, FZ, PH, JW, and HL contributed to the analysis and interpretation of data. All authors contributed to the article and approved the submitted version.

## Funding

This work was supported by the China MOST2030 Brain Project No. 2022ZD0208500 and the National Natural Science Foundation of China (grant numbers: 61871420; 62171101). The funding sources had no role in the design, methods, subject recruitment, data collection, analysis, or preparation of the paper.

## Conflict of interest

The authors declare that the research was conducted in the absence of any commercial or financial relationships that could be construed as a potential conflict of interest.

## Publisher’s note

All claims expressed in this article are solely those of the authors and do not necessarily represent those of their affiliated organizations, or those of the publisher, the editors and the reviewers. Any product that may be evaluated in this article, or claim that may be made by its manufacturer, is not guaranteed or endorsed by the publisher.

## Footnotes

^1^ https://central.xnat.org

^2^ http://rfmri.org/DPARSF

^3^ http://www.fil.ion.ucl.ac.uk/spm/software/spm12

^4^ http://rfmri.org/dpabi

^5^ http://dbm.neuro.uni-jena.de/vbm/

^6^ https://www.imaios.com/en/e-Anatomy/Anatomical-Parts/Paracentral-lobule

## References

[ref1] Abramian D. Larsson M. Eklund A. Aganj I. Westin C. F. Behjat H. (2021). Diffusion-informed spatial smoothing of fMRI data in white matter using spectral graph filters. NeuroImage 237:118095. doi: 10.1016/j.neuroimage.2021.118095, PMID: 34000402 PMC8356807

[ref2] Abramian D. Larsson M. Eklund A. Behjat H. (2020). Improved Functional Mri Activation Mapping in White Matter through Diffusion-Adapted Spatial Filtering. In *2020 Ieee 17th International Symposium on Biomedical Imaging (Isbi 2020)* Vol 237, 118095.

[ref3] Alexopoulos P. Sorg C. Forschler A. Grimmer T. Skokou M. Wohlschlager A.. (2012). Perfusion abnormalities in mild cognitive impairment and mild dementia in Alzheimer's disease measured by pulsed arterial spin labeling MRI. Eur. Arch. Psychiatry Clin. Neurosci. 262, 69–77. doi: 10.1007/s00406-011-0226-2, PMID: 21786091

[ref4] Bai F. Liao W. Watson D. R. Shi Y. M. Wang Y. Yue C. X.. (2011). Abnormal whole-brain functional connection in amnestic mild cognitive impairment patients. Behav. Brain Res. 216, 666–672. doi: 10.1016/j.bbr.2010.09.010, PMID: 20851147

[ref5] Bao J. F. Tu H. Li Y. J. Sun J. B. Hu Z. G. Zhang F. S.. (2022). Diffusion tensor imaging revealed microstructural changes in Normal-appearing white matter regions in relapsing-remitting multiple sclerosis. Front. Neurosci. 16. doi: 10.3389/fnins.2022.837452, PMID: 35310094 PMC8924457

[ref6] Barbas H. (2007). Flow of information for emotions through temporal and orbitofrontal pathways. J. Anat. 211, 237–249. doi: 10.1111/j.1469-7580.2007.00777.x, PMID: 17635630 PMC2375774

[ref7] Bellebaum C. Koch B. Schwarz M. Daum I. (2008). Focal basal ganglia lesions are associated with impairments in reward-based reversal learning. Brain 131, 829–841. doi: 10.1093/brain/awn011, PMID: 18263624

[ref8] Beuriat P. A. Cohen-Zimerman S. Smith G. N. L. Krueger F. Gordon B. Grafman J. (2020). A new insight on the role of the cerebellum for executive functions and emotion processing in adults. Front. Neurol. 11:593490. doi: 10.3389/fneur.2020.593490, PMID: 33424746 PMC7786249

[ref9] Biswal B. Yetkin F. Z. Haughton V. M. Hyde J. S. (1995). Functional connectivity in the motor cortex of resting human brain using Echo-planar Mri. Magn. Reson. Med. 34, 537–541. doi: 10.1002/mrm.1910340409 8524021

[ref10] Brun A. Englund E. (1986). A white matter disorder in dementia of the Alzheimer type: a pathoanatomical study. Ann. Neurol. 19, 253–262. doi: 10.1002/ana.410190306, PMID: 3963770

[ref11] Buckner R. L. Andrews-Hanna J. R. Schacter D. L. (2008). The brain's default network—anatomy, function, and relevance to disease. Year Cognit. Neurosci. 1124, 1–38. doi: 10.1196/annals.1440.011 18400922

[ref12] Cavada C. Company T. Tejedor J. Cruz-Rizzolo R. J. Reinoso-Suarez F. (2000). The anatomical connections of the macaque monkey orbitofrontal cortex. A review. Cereb. Cortex 10, 220–242. doi: 10.1093/cercor/10.3.220, PMID: 10731218

[ref13] Chandra A. Dervenoulas G. Politis M. Alzheimer's Disease Neuroimaging, I (2019). Magnetic resonance imaging in Alzheimer's disease and mild cognitive impairment. J. Neurol. 266, 1293–1302. doi: 10.1007/s00415-018-9016-3, PMID: 30120563 PMC6517561

[ref14] Chen J. J. Pike G. B. (2009). BOLD-specific cerebral blood volume and blood flow changes during neuronal activation in humans. NMR Biomed. 22, 1054–1062. doi: 10.1002/nbm.1411, PMID: 19598180

[ref15] Chen G. Ward B. D. Chen G. Li S. J. (2014). Decreased effective connectivity from cortices to the right parahippocampal gyrus in Alzheimer's disease subjects. Brain Connect. 4, 702–708. doi: 10.1089/brain.2014.0295 25132215 PMC4238239

[ref16] Chen Y. C. Xia W. Q. Luo B. Muthaiah V. P. K. Xiong Z. Y. Zhang J.. (2015). Frequency-specific alternations in the amplitude of low-frequency fluctuations in chronic tinnitus. Front. Neural Circuits 9, 00067. doi: 10.3389/fncir.2015.00067, PMID: 26578894 PMC4624866

[ref17] Chong J. S. X. Liu S. W. Loke Y. M. Hilal S. Ikram M. K. Xu X.. (2017). Influence of cerebrovascular disease on brain networks in prodromal and clinical Alzheimer's disease. Brain 140, 3012–3022. doi: 10.1093/brain/awx224, PMID: 29053778 PMC5841199

[ref18] Dahlin E. Neely A. S. Larsson A. Backman L. Nyberg L. (2008). Transfer of learning after updating training mediated by the striatum. Science 320, 1510–1512. doi: 10.1126/science.1155466, PMID: 18556560

[ref19] de Jong L. W. van der Hiele K. Veer I. M. Houwing J. J. Westendorp R. G. Bollen E. L.. (2008). Strongly reduced volumes of putamen and thalamus in Alzheimer's disease: an MRI study. Brain 131, 3277–3285. doi: 10.1093/brain/awn278, PMID: 19022861 PMC2639208

[ref20] de Leon M. J. George A. E. Golomb J. Tarshish C. Convit A. Kluger A.. (1997). Frequency of hippocampal formation atrophy in normal aging and Alzheimer's disease. Neurobiol. Aging 18, 1–11. doi: 10.1016/S0197-4580(96)00213-8 8983027

[ref21] Defrancesco M. Egger K. Marksteiner J. Esterhammer R. Hinterhuber H. Deisenhammer E. A.. (2014). Changes in white matter integrity before conversion from mild cognitive impairment to Alzheimer's disease. PLoS One 9:e106062. doi: 10.1371/journal.pone.0106062, PMID: 25153085 PMC4143363

[ref22] Di X. Kim E. H. Huang C. C. Tsai S. J. Lin C. P. Biswal B. B. (2013). The influence of the amplitude of low-frequency fluctuations on resting-state functional connectivity. Front. Hum. Neurosci. 7: 00118. doi: 10.3389/fnhum.2013.00118, PMID: 23565090 PMC3613753

[ref23] Dickerson B. C. Eichenbaum H. (2010). The episodic memory system: neurocircuitry and disorders. Neuropsychopharmacology 35, 86–104. doi: 10.1038/npp.2009.126, PMID: 19776728 PMC2882963

[ref24] Ding Z. Huang Y. Bailey S. K. Gao Y. Cutting L. E. Rogers B. P.. (2018). Detection of synchronous brain activity in white matter tracts at rest and under functional loading. Proc. Natl. Acad. Sci. U. S. A. 115, 595–600. doi: 10.1073/pnas.1711567115, PMID: 29282320 PMC5776967

[ref25] Ding Z. H. Xu R. Bailey S. K. Wu T. L. Morgan V. L. Cutting L. E.. (2016). Visualizing functional pathways in the human brain using correlation tensors and magnetic resonance imaging. Magn. Reson. Imaging 34, 8–17. doi: 10.1016/j.mri.2015.10.003, PMID: 26477562 PMC4714593

[ref26] Duarte A. Hayasaka S. Du A. T. Schuff N. Jahng G. H. Kramer J.. (2006). Volumetric correlates of memory and executive function in normal elderly, mild cognitive impairment and Alzheimer's disease. Neurosci. Lett. 406, 60–65. doi: 10.1016/j.neulet.2006.07.029, PMID: 16904823 PMC1779764

[ref27] Eichenbaum H. Sauvage M. Fortin N. Komorowski R. Lipton P. (2012). Towards a functional organization of episodic memory in the medial temporal lobe. Neurosci. Biobehav. Rev. 36, 1597–1608. doi: 10.1016/j.neubiorev.2011.07.006, PMID: 21810443 PMC3227798

[ref28] Fan L. Hu J. Ma W. Y. Wang D. H. Yao Q. Shi J. P. (2019). Altered baseline activity and connectivity associated with cognitive impairment following acute cerebellar infarction: a resting-state fMRI study. Neurosci. Lett. 692, 199–203. doi: 10.1016/j.neulet.2018.11.007, PMID: 30439397

[ref29] Ferreira L. K. Diniz B. S. Forlenza O. V. Busatto G. F. Zanetti M. V. (2011). Neurostructural predictors of Alzheimer's disease: a meta-analysis of VBM studies. Neurobiol. Aging 32, 1733–1741. doi: 10.1016/j.neurobiolaging.2009.11.008, PMID: 20005012

[ref30] Folstein M. F. Folstein S. E. McHugh P. R. (1975). Mini-mental state. A practical method for grading the cognitive state of patients for the clinician. J. Psychiatr. Res. 12, 189–198. doi: 10.1016/0022-3956(75)90026-6 1202204

[ref31] Fox M. D. Raichle M. E. (2007). Spontaneous fluctuations in brain activity observed with functional magnetic resonance imaging. Nat. Rev. Neurosci. 8, 700–711. doi: 10.1038/nrn2201 17704812

[ref32] Gawryluk J. R. Mazerolle E. L. D'Arcy R. C. (2014). Does functional MRI detect activation in white matter? A review of emerging evidence, issues, and future directions. Front. Neurosci. 8:239. doi: 10.3389/fnins.2014.00239 25152709 PMC4125856

[ref33] Gore J. C. Li M. Gao Y. Wu T. L. Schilling K. G. Huang Y.. (2019). Functional MRI and resting state connectivity in white matter—a mini-review. Magn. Reson. Imaging 63, 1–11. doi: 10.1016/j.mri.2019.07.017, PMID: 31376477 PMC6861686

[ref34] Greicius M. D. Srivastava G. Reiss A. L. Menon V. (2004). Default-mode network activity distinguishes Alzheimer's disease from healthy aging: evidence from functional MRI. Proc. Natl. Acad. Sci. U. S. A. 101, 4637–4642. doi: 10.1073/pnas.0308627101 15070770 PMC384799

[ref35] Guo X. Wang Z. Li K. Li Z. Qi Z. Jin Z.. (2010). Voxel-based assessment of gray and white matter volumes in Alzheimer's disease. Neurosci. Lett. 468, 146–150. doi: 10.1016/j.neulet.2009.10.086, PMID: 19879920 PMC2844895

[ref36] Gupta Y. Kim J. I. Kim B. C. Kwon G. R. Initia A. D. N. (2020). Classification and graphical analysis of Alzheimer's disease and its prodromal stage using multimodal features from structural, diffusion, and functional neuroimaging data and the APOE genotype. Front. Aging Neurosci. 12:00238. doi: 10.3389/fnagi.2020.00238, PMID: 32848713 PMC7406801

[ref37] Han Y. Wang J. H. Zhao Z. L. Min B. Q. Lu J. Li K. C.. (2011). Frequency-dependent changes in the amplitude of low-frequency fluctuations in amnestic mild cognitive impairment: a resting-state fMRI study. NeuroImage 55, 287–295. doi: 10.1016/j.neuroimage.2010.11.059, PMID: 21118724

[ref38] Harrington C. R. (2012). The molecular pathology of Alzheimer's disease. Neuroimaging Clin. N. Am. 22, 11–22. doi: 10.1016/j.nic.2011.11.003 22284730

[ref39] Helenius J. Perkiö J. Soinne L. Østergaard L. Carano R. A. D. Salonen O.. (2003). Cerebral HEMODYNAMICS in a healthy population measured by dynamic susceptibility contrast MR imaging. Acta Radiol. 44, 538–546. doi: 10.1080/j.1600-0455.2003.00104.x, PMID: 14510762

[ref40] Honey C. J. Sporns O. Cammoun L. Gigandet X. Thiran J. P. Meuli R.. (2009). Predicting human resting-state functional connectivity from structural connectivity. Proc. Natl. Acad. Sci. U. S. A. 106, 2035–2040. doi: 10.1073/pnas.0811168106, PMID: 19188601 PMC2634800

[ref41] Huang Y. L. Bailey S. K. Wang P. G. Cutting L. E. Gore J. C. Ding Z. H. (2018). Voxel-wise detection of functional networks in white matter. NeuroImage 183, 544–552. doi: 10.1016/j.neuroimage.2018.08.049, PMID: 30144573 PMC6226032

[ref42] Jaroudi W. Garami J. Garrido S. Hornberger M. Keri S. Moustafa A. A. (2017). Factors underlying cognitive decline in old age and Alzheimer's disease: the role of the hippocampus. Rev. Neurosci. 28, 705–714. doi: 10.1515/revneuro-2016-0086, PMID: 28422707

[ref43] Ji G.-J. Liao W. Chen F.-F. Zhang L. Wang K. (2017). Low-frequency blood oxygen level-dependent fluctuations in the brain white matter: more than just noise. Sci. Bull. 62, 656–657. doi: 10.1016/j.scib.2017.03.021, PMID: 36659309

[ref44] Kim J. Lee J. H. (2013). Integration of structural and functional magnetic resonance imaging improves mild cognitive impairment detection. Magn. Reson. Imaging 31, 718–732. doi: 10.1016/j.mri.2012.11.009, PMID: 23260395

[ref45] Kringelbach M. L. (2005). The human orbitofrontal cortex: linking reward to hedonic experience. Nat. Rev. Neurosci. 6, 691–702. doi: 10.1038/nrn1747, PMID: 16136173

[ref46] Lavenex P. Amaral D. G. (2000). Hippocampal-neocortical interaction: a hierarchy of associativity. Hippocampus 10, 420–430. doi: 10.1002/1098-1063(2000)10:4<420::Aid-Hipo8>3.3.Co;2-X 10985281

[ref47] Li J. Biswal B. B. Wang P. Duan X. Cui Q. Chen H.. (2019). Exploring the functional connectome in white matter. Hum. Brain Mapp. 40, 4331–4344. doi: 10.1002/hbm.24705, PMID: 31276262 PMC6865787

[ref48] Li X. Coyle D. Maguire L. Watson D. R. McGinnity T. M. (2011). Gray matter concentration and effective connectivity changes in Alzheimer's disease: a longitudinal structural MRI study. Neuroradiology 53, 733–748. doi: 10.1007/s00234-010-0795-1 21113707

[ref49] Liu W. Liu L. Cheng X. X. Ge H. L. Hu G. J. Xue C.. (2021). Functional integrity of executive control network contributed to retained executive abilities in mild cognitive impairment. Front. Aging Neurosci. 13:710172. doi: 10.3389/fnagi.2021.710172, PMID: 34899264 PMC8664557

[ref50] Liu J. H. Yin C. H. Xia S. G. Jia L. F. Guo Y. Q. Zhao Z. L.. (2013). White matter changes in patients with amnestic mild cognitive impairment detected by diffusion tensor imaging. PLoS One 8:e59440. doi: 10.1371/journal.pone.0059440, PMID: 23555673 PMC3605411

[ref51] Luo F. F. Wang J. B. Yuan L. X. Zhou Z. W. Xu H. Ma S. H.. (2020). Higher sensitivity and reproducibility of wavelet-based amplitude of resting-state fMRI. Front. Neurosci. 14:00224. doi: 10.3389/fnins.2020.00224, PMID: 32300288 PMC7145399

[ref52] Mak M. Tyburski E. Madany L. Sokolowski A. Samochowiec A. (2016). Executive function deficits in patients after cerebellar neurosurgery. J. Int. Neuropsychol. Soc. 22, 47–57. doi: 10.1017/S1355617715001174, PMID: 26626541

[ref53] Mazerolle E. L. Ohlhauser L. Mayo C. D. Sheriff A. Gawryluk J. R. (2020). Evidence of underreporting of white matter fMRI activation. J. Magn. Reson. Imaging 51, 1596–1597. doi: 10.1002/jmri.26952, PMID: 31667922

[ref54] McKhann G. M. Knopman D. S. Chertkow H. Hyman B. T. Jack C. R. Jr. Kawas C. H.. (2011). The diagnosis of dementia due to Alzheimer's disease: recommendations from the National Institute on Aging-Alzheimer's Association workgroups on diagnostic guidelines for Alzheimer's disease. Alzheimers Dement. 7, 263–269. doi: 10.1016/j.jalz.2011.03.005, PMID: 21514250 PMC3312024

[ref55] Morris J. C. (1993). The clinical dementia rating (Cdr)—current version and scoring rules. Neurology 43, 2412–2414. doi: 10.1212/WNL.43.11.2412-a, PMID: 8232972

[ref56] Myers P. S. McNeely M. E. Koller J. M. Earhart G. M. Campbell M. C. (2017). Cerebellar volume and executive function in Parkinson disease with and without freezing of gait. J. Parkinsons Dis. 7, 149–157. doi: 10.3233/Jpd-161029, PMID: 28106569 PMC5322196

[ref57] Pennanen C. Kivipelto M. Tuomainen S. Hartikainen P. Hanninen T. Laakso M. P.. (2004). Hippocampus and entorhinal cortex in mild cognitive impairment and early AD. Neurobiol. Aging 25, 303–310. doi: 10.1016/S0197-4580(03)00084-8, PMID: 15123335

[ref58] Petersen R. C. Doody R. Kurz A. Mohs R. C. Morris J. C. Rabins P. V.. (2001). Current concepts in mild cognitive impairment. Arch. Neurol. 58, 1985–1992. doi: 10.1001/archneur.58.12.1985 11735772

[ref59] Power J. D. Barnes K. A. Snyder A. Z. Schlaggar B. L. Petersen S. E. (2012). Spurious but systematic correlations in functional connectivity MRI networks arise from subject motion. NeuroImage 59, 2142–2154. doi: 10.1016/j.neuroimage.2011.10.018 22019881 PMC3254728

[ref60] Ronald C. Petersen P. Doody R. Kurz A. Mohs R. C. Morris J. C.. (2001). Current Concepts in Mild Cognitive Impairment. Arch Neurol, 58: 1985–1992. doi: 10.1001/archneur.58.12.1985 11735772

[ref61] Roquet D. Noblet V. Anthony P. Philippi N. Demuynck C. Cretin B.. (2017). Insular atrophy at the prodromal stage of dementia with Lewy bodies: a VBM DARTEL study. Sci. Rep. 7:9437. doi: 10.1038/s41598-017-08667-7, PMID: 28842567 PMC5573371

[ref62] Rosales-Corral S. A. Acuna-Castroviejo D. Coto-Montes A. Boga J. A. Manchester L. C. Fuentes-Broto L.. (2012). Alzheimer's disease: pathological mechanisms and the beneficial role of melatonin. J. Pineal Res. 52, 167–202. doi: 10.1111/j.1600-079X.2011.00937.x, PMID: 22107053

[ref63] Rostrup E. Law I. Blinkenberg M. Larsson H. B. Born A. P. Holm S.. (2000). Regional differences in the CBF and BOLD responses to hypercapnia: a combined PET and fMRI study. NeuroImage 11, 87–97. doi: 10.1006/nimg.1999.0526, PMID: 10679182

[ref64] Schmahmann J. D. (1991). An emerging concept—the cerebellar contribution to higher function. Arch. Neurol. 48, 1178–1187. doi: 10.1001/archneur.1991.00530230086029 1953406

[ref65] Sorg C. Riedl V. Muhlau M. Calhoun V. D. Eichele T. Laer L.. (2007). Selective changes of resting-state networks in individuals at risk for Alzheimer's disease. Proc. Natl. Acad. Sci. U. S. A. 104, 18760–18765. doi: 10.1073/pnas.0708803104, PMID: 18003904 PMC2141850

[ref66] Spaniol J. Davidson P. S. R. Kim A. S. N. Han H. Moscovitch M. Grady C. L. (2009). Event-related fMRI studies of episodic encoding and retrieval: Meta-analyses using activation likelihood estimation. Neuropsychologia 47, 1765–1779. doi: 10.1016/j.neuropsychologia.2009.02.028, PMID: 19428409

[ref67] Teipel S. J. Born C. Ewers M. Bokde A. L. W. Reiser M. F. Moller H. J.. (2007). Multivariate deformation-based analysis of brain atrophy to predict Alzheimer's disease in mild cognitive impairment. NeuroImage 38, 13–24. doi: 10.1016/j.neuroimage.2007.07.008, PMID: 17827035

[ref68] Tisserand D. J. van Boxtel M. P. J. Pruessner J. C. Hofman P. Evans A. C. Jolles J. (2004). A voxel-based morphometric study to determine individual differences in gray matter density associated with age and cognitive change over time. Cereb. Cortex 14, 966–973. doi: 10.1093/cercor/bhh057, PMID: 15115735

[ref69] Van Hoesen G. W. Augustinack J. C. Dierking J. Redman S. J. Thangavel R. (2000). The parahippocampal gyrus in Alzheimer's disease—clinical and preclinical neuroanatomical correlates. Parahippocampal Region 911, 254–274. doi: 10.1111/j.1749-6632.2000.tb06731.x 10911879

[ref70] Vasavada M. M. Wang J. L. Eslinger P. J. Gill D. J. Sun X. Y. Karunanayaka P.. (2015). Olfactory cortex degeneration in Alzheimer's disease and mild cognitive impairment. J. Alzheimers Dis. 45, 947–958. doi: 10.3233/Jad-141947 25633674

[ref71] Wagner M. J. Luo L. Q. (2020). Neocortex-cerebellum circuits for cognitive processing. Trends Neurosci. 43, 42–54. doi: 10.1016/j.tins.2019.11.002, PMID: 31787351 PMC6942222

[ref72] Wang Z. J. Dai Z. J. Gong G. L. Zhou C. S. He Y. (2015). Understanding structural-functional relationships in the human brain: a large-scale network perspective. Neuroscientist 21, 290–305. doi: 10.1177/1073858414537560 24962094

[ref73] Wang L. Kong Q. M. Li K. Su Y. N. Zeng Y. W. Zhang Q. G.. (2016). Frequency-dependent changes in amplitude of low-frequency oscillations in depression: a resting-state fMRI study. Neurosci. Lett. 614, 105–111. doi: 10.1016/j.neulet.2016.01.012, PMID: 26797652

[ref74] Wang P. Meng C. Yuan R. Wang J. Yang H. Zhang T.. (2020). The organization of the human corpus callosum estimated by intrinsic functional connectivity with white-matter functional networks. Cereb. Cortex 30, 3313–3324. doi: 10.1093/cercor/bhz311, PMID: 32080708

[ref75] Wang P. J. Saykin A. J. Flashman L. A. Wishart H. A. Rabin L. A. Santulli R. B.. (2006). Regionally specific atrophy of the corpus callosum in AD, MCI and cognitive complaints. Neurobiol. Aging 27, 1613–1617. doi: 10.1016/j.neurobiolaging.2005.09.035, PMID: 16271806 PMC3482483

[ref76] Wang P. Wang J. Michael A. Wang Z. Klugah-Brown B. Meng C.. (2022). White matter functional connectivity in resting-state fMRI: robustness, reliability, and relationships to gray matter. Cereb. Cortex 32, 1547–1559. doi: 10.1093/cercor/bhab181, PMID: 34753176

[ref77] Wang P. Wang J. Tang Q. Alvarez T. L. Wang Z. Kung Y. C.. (2021). Structural and functional connectivity mapping of the human corpus callosum organization with white-matter functional networks. NeuroImage 227:117642. doi: 10.1016/j.neuroimage.2020.117642, PMID: 33338619

[ref78] Wang Z. Q. Yan C. G. Zhao C. Qi Z. G. Zhou W. D. Lu J.. (2011). Spatial patterns of intrinsic brain activity in mild cognitive impairment and Alzheimer's disease: a resting-state functional MRI study. Hum. Brain Mapp. 32, 1720–1740. doi: 10.1002/hbm.21140, PMID: 21077137 PMC6870362

[ref79] Weller J. Budson A. (2018). Current understanding of Alzheimer's disease diagnosis and treatment. F1000Res 7:1161. doi: 10.12688/f1000research.14506.1, PMID: 30135715 PMC6073093

[ref80] Witter M. P. Naber P. A. van Haeften T. Machielsen W. C. Rombouts S. A. Barkhof F.. (2000). Cortico-hippocampal communication by way of parallel parahippocampal-subicular pathways. Hippocampus 10, 398–410. doi: 10.1002/1098-1063(2000)10:4<398::AID-HIPO6>3.0.CO;2-K, PMID: 10985279

[ref81] Wu X. Yang Z. P. Bailey S. K. Zhou J. L. Cutting L. E. Gore J. C.. (2017). Functional connectivity and activity of white matter in somatosensory pathways under tactile stimulations. NeuroImage 152, 371–380. doi: 10.1016/j.neuroimage.2017.02.074, PMID: 28284801 PMC5432381

[ref82] Yang L. Yan Y. Wang Y. H. Hu X. C. Lu J. Chan P.. (2018). Gradual disturbances of the amplitude of low-frequency fluctuations (ALFF) and fractional ALFF in Alzheimer Spectrum. Front. Neurosci. 12:00975. doi: 10.3389/fnins.2018.00975, PMID: 30618593 PMC6306691

[ref83] Zang Y. F. He Y. Zhu C. Z. Cao Q. J. Sui M. Q. Liang M.. (2012). Erratum to “altered baseline brain activity in children with ADHD revealed by resting-state functional MRI” [brain develop 29 (2) (2007) 83–91]. Brain Dev. 34:336. doi: 10.1016/j.braindev.2012.01.002 16919409

